# Outpatient care in acute and prehospital emergency medicine by emergency medical and patient transport service over a 10-year period: a retrospective study based on dispatch data from a German emergency medical dispatch centre (OFF-RESCUE)

**DOI:** 10.1186/s12873-021-00424-4

**Published:** 2021-03-09

**Authors:** Marc S. Schehadat, Guido Scherer, David A. Groneberg, Manfred Kaps, Michael H. K. Bendels

**Affiliations:** 1 Institute for Occupational Medicine, Social Medicine and Environmental Medicine, University Hospital Frankfurt, Theodor-Stern-Kai 7, House 9b, 60590 Frankfurt/Main, Germany; 2 Department of Neurology, University Hospital Giessen and Marburg, Giessen, Germany; 3 District Administration Mainz-Bingen, Department of Civil Protection, Ingelheim/Rhein, Germany

**Keywords:** OFF-Mission, ON-Mission, Emergency medical control center, Triaged, Dispatched, Rural, Urban, Job cycle time, Prehospital emergency care, Emergency medical dispatch

## Abstract

**Background:**

The number of operations by the German emergency medical service almost doubled between 1994 and 2016. The associated expenses increased by 380% in a similar period. Operations with treatment on-site, which retrospectively proved to be misallocated (OFF-Missions), have a substantial proportion of the assignment of the emergency medical service (EMS). Besides OFF-Missions, operations with patient transport play a dominant role (named as ON-Missions). The aim of this study is to work out the medical and economic relevance of both operation types.

**Methods:**

This analysis examined *N* = 819,780 missions of the EMS and patient transport service (PTS) in the catchment area of the emergency medical dispatch centre (EMDC) Bad Kreuznach over the period from 01/01/2007 to 12/31/2016 in terms of triage and disposition, urban-rural distribution, duration of operations and economic relevance (*p* < .01).

**Results:**

53.4% of ON-Missions are triaged with the indication non-life-threatening patient transport; however, 63.7% are processed by the devices of the EMS. Within the OFF-Mission cohort, 78.2 and 85.8% are triaged or dispatched for the EMS. 74% of all ON-Missions are located in urban areas, 26% in rural areas; 81.3% of rural operations are performed by the EMS. 66% of OFF-Missions are in cities. 93.2% of the remaining 34% of operations in rural locations are also performed by the EMS. The odds for both ON- and OFF-Missions in rural areas are significantly higher than for PTS (OR_ON_ 3.6, 95% CI 3.21–3.30; OR_OFF_ 3.18, 95% CI 3.04–3.32). OFF-Missions last 47.2 min (SD 42.3; CI 46.9–47.4), while ON-Missions are processed after 79.7 min on average (SD 47.6; CI 79.6–79.9). ON-Missions generated a turnover of more than € 114 million, while OFF-Missions made a loss of almost € 13 million.

**Conclusions:**

This study particularly highlights the increasing utilization of emergency devices; especially in OFF-Missions, the resources of the EMS have a higher number of operations than PTS. OFF-Missions cause immensely high costs due to misallocations from an economic point of view. Appropriate patient management appears necessary from both medical and economic perspective, which requires multiple solution approaches.

## Background

The utilization of the resources of the emergency medical service (EMS) and patient transport service (PTS) in the public healthcare system has become increasingly important in recent years [1–3]; while in Germany in 1994 about 9.5 million operations were performed, in 2016 there were already 16.4 million missions [4]. In retrospect, most missions (70%) were non-urgent and mainly took place in cities [5–8]. In particular, the number of *outpatient medical contacts* in emergency medicine accounted for a significant proportion; this was rated as 16–41.7% [9–13]. Here, patients often misjudged the urgency of their complaints [14]. This mainly concerned vulnerable patient groups such as elders, children, young adults and homeless people; neurological and traumatological indications were frequent reasons for emergency calls; in this context, the information in the relevant literature for neurological (1–29%) and traumatological complaints such as injuries caused by falls (9–56%) vary [15–18].

A distinction is made between assistance services, where a medical service is performed on-site, and operations, where no patient is present at the emergency location [19]. Statistically, spurious trips are recorded as a quota by the German Federal Health Monitoring; in 2013, a proportion of 7.4% of all operations of the public EMS was documented [19]. In contrast to patient transportations, assistance services and spurious trips are not remunerated in Rhineland-Palatinate; these costs must be cross-financed by the organizations that run the EMS; they are included in the operating expenses [20]. Assistant services are not officially recorded by statistics. In order to reduce these non-life-threatening operations in favour of efficient patient management, various solution approaches have been applied in the past; processes of the emergency medical dispatch centre (EMDC) were improved in respect of emergency call handling, which, however, led to an over-prioritization (false positive assignment of a higher priority level) of many patients [21–23]. In addition, protocols for handling low-priority patients have been successfully implemented, which lead either to the outpatient usage of a nurse or to telephone consultation [24, 25]. In general, the decision on whether a patient should be conveyed at all is made by the team on scene depending on the paramedic’s qualification which is different in EMS and PTS; lower qualified paramedics mainly work in PTS, while higher qualified paramedics work in EMS. The reason is that in the rescue service area studied, the PTS is almost used exclusively for so-called appointment trips (e.g., trips to medical specialists), in which there has already been contact with a physician (e.g., general practitioner) and it was not an emergency call. The EMS mainly provides assistance for so-called primary missions, in which no physician was on the scene and assessed the patient’s condition. So far, several studies have shown inconsistent findings that the paramedic on site is able to decide due to a lack of patient safety whether a patient should be conveyed [26–28]. Participation of general practitioners in prehospital emergency care would help [29]. During non-indicated operations, emergency devices are not available for life-threatening patients, which delays the ambulance response time. As a result, the outcome of patients with time-critical emergency gets worse [30–32].

A similar development of overuse is also apparent in the emergency departments [33]. Important reasons are on the one hand the lack of awareness of medical necessity and on the other hand the fact that young people in particular see the emergency department as a contact point outside the opening hours of general practitioners as a substitute service [34–36]. For most patients (66%), outpatient medical care is sufficient, but it still caused an immense effort [37]. From an economic point of view, healthcare costs in Germany increased by 236% from about € 159 to € 375 billion between 1992 and 2017. The EMS even registered a rise of 380%, the share of healthcare costs rose from 0.8 to 1.3% [38].

In the following, the term OFF-Mission will describe all operations that did not prehospitally result in a patient transportation, while ON-Missions describe completed transports. The aim of this retrospective, explorative study is to compare ON- and OFF-Missions in terms of urban-rural distribution, job cycle time and economic relevance.

### Current procedure for the prioritization of outpatient emergency missions

In Germany, all medical emergencies should be reported via the European emergency number 112 to the responsible EMDC, which organizes the non-police emergency response. In the rescue service area Bad Kreuznach/Rhineland-Palatinate, patient transports can be requested via the same institution, but also under the service number 19222. The emergency call processing at the EMDC is performed in several steps; at first, the call handler prioritizes the urgency of the request based on his initial assessment, which is followed by the decision on the appropriate device (*Triaged*). A distinction is made between emergency devices such as emergency rescue vehicles, which are primarily responsible for emergency events, and patient transport vehicle, which carry out adaptable transports, e.g. to planned medical examinations. In the next step, the so-called dispatcher may be forced to dispatch an inadequate device due to organizational limitations regarding the lack of availability of correct resources and by consideration of the entire overall scenario (*Dispatched*). In general, the most important principle is the fastest possible dispatch of a device to a life-threatening patient; in daily practice, this means that an immediate available patient transport vehicle with medical resources may can provide assistance more quickly than an emergency rescue vehicle with extensive diagnostic and therapeutic facilities that is still tied up in an operation, because its time availability cannot be estimated at the time the emergency call is received.

## Methods

### Data acquisition

One of the eight EMDCs in Rhineland-Palatinate/Germany, located in Bad Kreuznach, was selected for the data collection. This EMDC is responsible for the three districts Bad Kreuznach, Birkenfeld and Rhine-Hunsrück with 341,215 inhabitants, which represent 8.4% of the total population of the federal state and an area of 2631.88 km^2^ with a proportion of 13.3% of the territory of Rhineland-Palatinate [39]. To classify between city and rural municipality, only the status given by the government was considered.

For this research the following variables were used from the SQL database: radio coding of the devices, emergency scene by location, emergency scene by object from any existing database entry, type of operation, operation keyword, diagnosis, comments, type of transport, first operation code (corresponds to indication), short comment of first operation code, second operation code, short comment of second operation code, third operation code, short comment of third operation code, caller, destination, destination district, operation opening time, takeover time, end time of operation, time of alerting, job cycle time.

### Study type

**Fig. 1:**
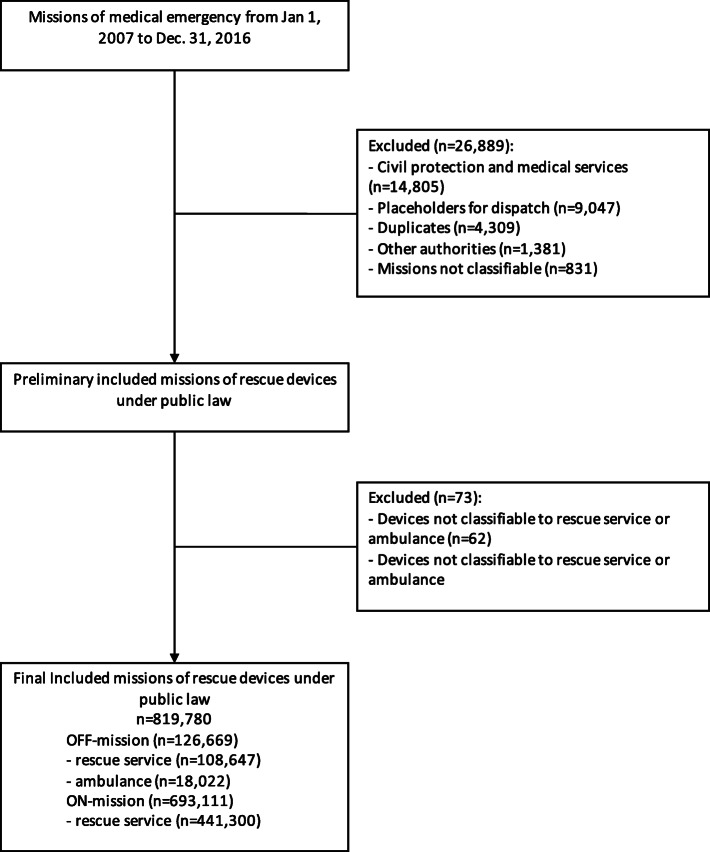
Study Flow Chart. Study Flow Chart with inclusion and exclusion criteria

This study is a descriptive analysis. In the observation period from 01/01/2007 to 12/31/2016, *N* = 819,780 ON- and OFF-Missions of the public emergency medical and patient transport service in the catchment area of the EMDC Bad Kreuznach were included. All operations that could not be clearly assigned to a type of transport or prehospital device, were test operations, duplicates or placeholders and were performed either by civil protection or first responder were excluded (see Fig. 1).

### Dispatching types

During the investigation period, two different types of dispatching could be distinguished due to a change in the control center software: from 2007 to 2011, the selection of the correct device based on emergency and operation categories such as *internal emergency*. From 2012 to 2016, the disposition procedure was performed according to the so-called operation codes (EC), which code both symptoms/ suspected diagnoses and organizational indications, such as *acute stroke* or *technical rescue*. This resulted, among other things, in the transport types *R* for an urgent operation in an emergency rescue vehicle or *KL* for a non-urgent transport in horizontal position in a patient transport vehicle. For this reason, the query script had to contain the old and current operation indications.

### Remuneration

The fees for operations of the EMS corresponded to the level of 2016 and were used as a basis for the 10-year period investigated. Extra charges for nights, Sundays and public holidays as well as mileage and emergency physician’s flat rate and dispatch centre fees were not taken into account. At this point it should be mentioned that the performance of the emergency physician is always remunerated in the form of the emergency physician’s flat rate independent from the type of mission, i.e. ON- or OFF-Mission. The only condition is the presence of a living patient. Here, a simplified attempt will be made to determine the value of ON- and OFF-Missions or their lost revenues. The operating minute of the specific device was used as the basis for calculation.

### Data integration

The data sets collected were processed by using Microsoft Excel (Version 16.33, Redmond, WA/USA); subsequently they were anonymized, corrected and standardized.

### Statistical analysis

**Fig. 2:**
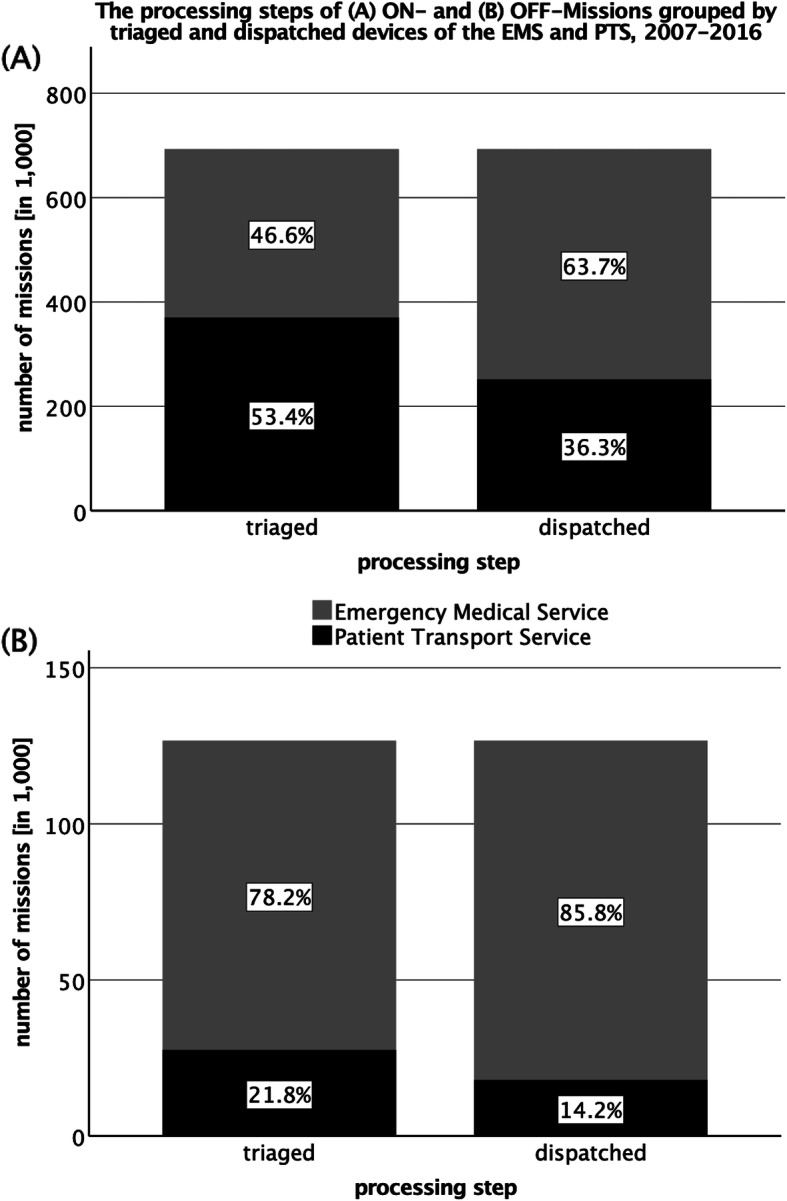
The processing steps of ON- and OFF-Missions grouped by triaged and dispatched devices of the EMS and PTS. 2007–2016. **a** Grouping of all ON-Missions of the EMS and PTS according to the processing steps Triaged and Dispatched, **b** corresponding grouping of all OFF-Missions. EMS Emergency Medical Service, PTS Patient Transport Service

**Fig. 3:**
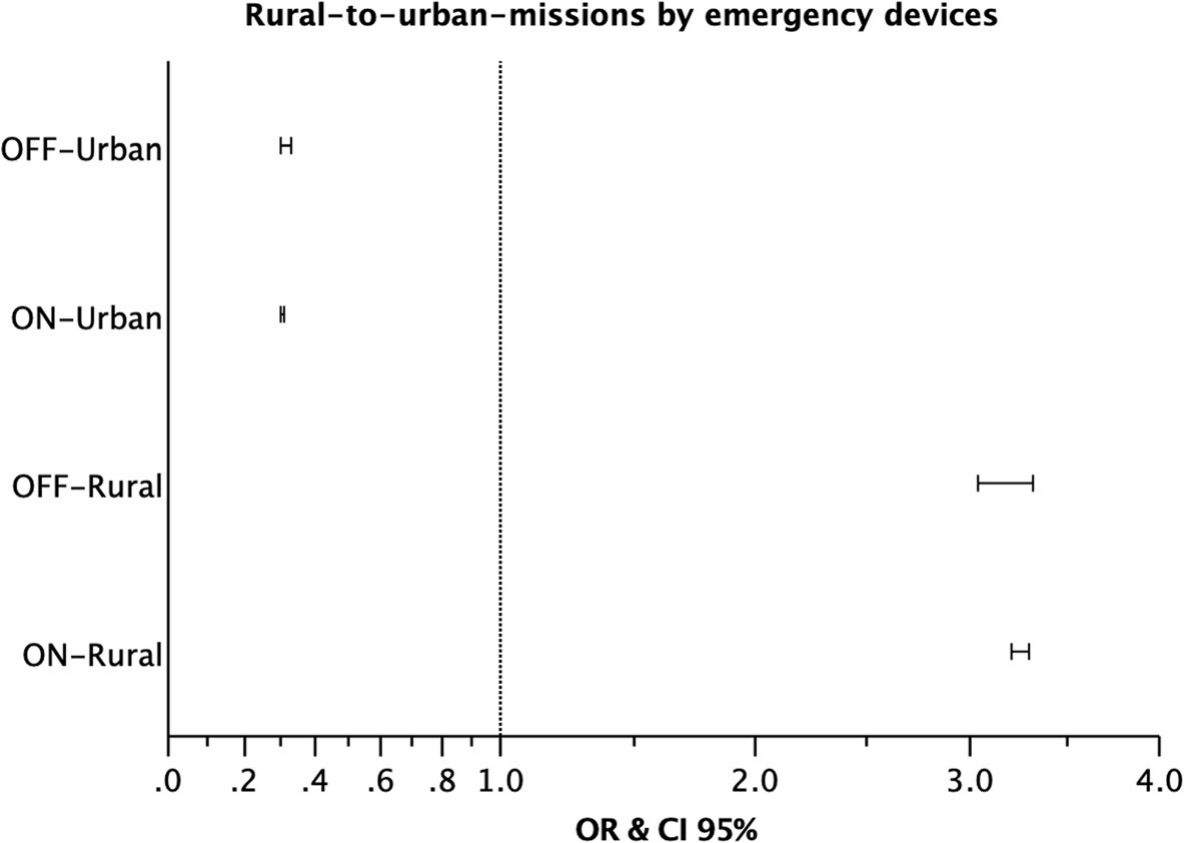
Rural-to-urban-missions by emergency devices. Odds ratios of ON- and OFF-Missions in urban and rural settings by emergency devices, 2007–2016. The odds of emergency devices for ON- and OFF-Missions in rural areas are significantly high

**Fig. 4:**
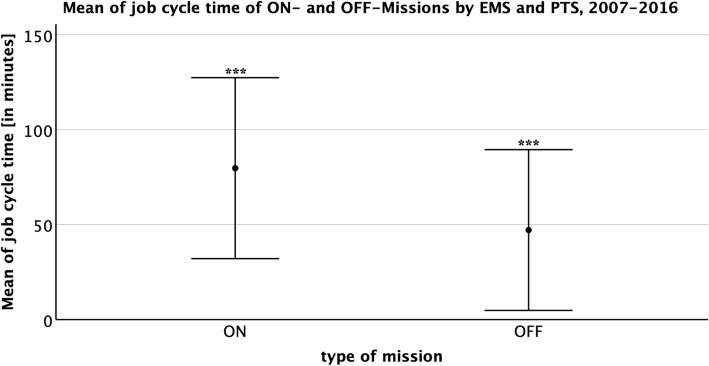
Mean of job cycle time of ON- and OFF-Missions by EMS and PTS, 2007–2016. Means of the job cycle time of ON- and OFF-Missions of the EMS and PTS in minutes from 2007 to 2016 with standard deviation. For the sake of clarity, the upper extreme values have been hidden

The independent samples t test was performed for Fig. 2 with the variables *EM-Typ* (corresponds to *Dispatched*) and *Einsatzart* (corresponds to *Triaged*), for Fig. 3 with *Ortsteil* (corresponds to *type of mission*) and *EM-Typ* and for Fig. 4 with *Gesamtdauer* (corresponds to *job cycle time*). The statistical significance level was set at *p* < .01. The relevant topics were analyzed statistically by using SPSS (Version 25, IBM Corp., Armonk, NY/USA).

## Results

### Triage of emergency calls and dispatching of resources

Among the ON-Missions, 53.4% (*N* = 370,394) of all telephone requests were for PTS, while the remaining 46.6% (*N* = 322,717) were classified as emergencies (Fig. 2a). Looking at the dispatched emergency devices, the resources of the EMS provided 63.7% (*N* = 441,300) of all operations, while PTS only provided 36.3% (*N* = 251,811). The triaged (78.2%; *N* = 99,033) and dispatched OFF-Missions (85.8%, *N* = 108,647) were predominantly assigned to the vehicles of the EMS (Fig. 2b). The proportion of the PTS is relatively small, both for triaged (21.8%; *N* = 27,636) and dispatched operations (14.2%; *N* = 18,022).

### Urban-rural distribution of ON- and OFF-missions

From 2007 to 2016, 693,111 ON- and 126,669 OFF-Missions were recorded. 68.5% of all operations took place in the city, 25.6% in rural areas and 6% in foreign or neighboring catchment areas. 74% of all ON-Missions were in urban emergency locations (*N* = 483,884); 57.1% were performed by EMS and 42.9% by PTS. The remaining 26% of all ON-Missions took place in a rural context (*N* = 169,727); EMS accounted for 81.3% and PTS for 18.8% of the operations. 66.1% of all OFF-Missions were in urban emergency locations (*N* = 77,519); whereby 81.2% were performed by emergency devices and 18.7% patient transport vehicles. The remaining 34% of all OFF-Missions took place in a rural context (*N* = 39,905); 93.2% were carried out by EMS and 6.8% by PTS. The analysis of the urban and rural distribution of operations did not include the missions of the devices of the EMDC Bad Kreuznach to emergency locations in neighboring catchment areas (N_foreign_ = 48,745, N_OFF_foreign_ = 7.3%, N_ON_foreign_ = 5.7%).

The odds of an ON-Mission in a rural location performed by an emergency vehicle were more than three times higher than by a patient transport vehicle (Fig. 3) (OR 3.26, 95% CI 3.21–3.30). This was similar for OFF-Missions (OR 3.18, 95% CI 3.04–3.32). In the urban setting it was vice versa; the probability of a patient transportation (OR 0.31, 95% CI 0.30–0.31) and an OFF-Mission by an emergency vehicle was smaller than by a patient transport vehicle (OR 0.31, 95% CI 0.30–0.33).

### Job cycle time of ON- and OFF-missions

The total mean of the job cycle time of both mission classifications was 74.7 min (SD = 42.3, CI 46.9–47.4) (Fig. 4). The OFF-Missions had a smaller mean of 47.2 min (SD 42.3; CI 46.9–47.4) compared to the ON-Missions, which lasted 79.7 min (SD 47.6; CI 79.6–79.9). In both operation cohorts, the shortest missions were reported to last about 2 s and the longest about 24 h.

### Economic view

Assuming that the operation minute of an emergency rescue vehicle was worth € 3.50 at an average job cycle time of 79 min, the costs for emergency transports amounted to more than € 100 million for the period under investigation (Table 1). Based on this calculation and an average job cycle time of an OFF-Mission of approximately 44 min, the lost revenue per mission was € 155.40 (Table 2). With a total of 67,831 OFF-missions, this resulted in a deficit of more than € 10 million. Across all mission categories, this resulted in a total deficit of € 12,724,513, which corresponded to 11.1% of the turnover of all ON-Missions at € 114,506,436.

**Table 1:** Economic key figures of ON-missions in Euro according to the basic rates from 2016 by the devices of the EMS and PTS, 2007–2016

	Mean of job cycle time in minutes	Basic rate	Cost per minute	Total number	Total cost
Device					
Emergency rescue vehicle	78.7	278.8	3.5	371,853	103,654,024
Emergency physician operational vehicle	82.7	79.0	1.0	65,454	5,168,248
Patient transport vehicle	80.3	22.5	0.3	212,435	4,784,036
Urgent patient transport vehicle	78.6	22.5	0.3	39,376	886,748
Emergency physicians‘car	304.7	278.8	0.9	48	13,380

**Table 2:** Economic key figures of OFF-Missions in Euro according to the basic rates from 2016 by the devices of the EMS and PTS, 2007-2016

	Mean of job cycle time in minutes	Lost revenue per mission	Cost per minute	Total number	Total cost
Device					
Emergency rescue vehicle	43.9	155.4	3.5	67,831	10,543,651
Emergency physician operational vehicle	54	51.8	1.0	37,689	1,951,913
Patient transport vehicle	45.2	12.7	0.3	14,950	189,865
Urgent patient transport vehicle	43	12.5	0.3	3,072	38,400
Emergency physicians‘ car	107.4	97.7	0.9	7	684

## Discussion

### Triage and dispatching

The results of the ON-Missions show a clear disproportion in the triaging and disposition of PTS-induced operations (Fig. 2). 63.7% of all operations were performed by the EMS, although only 46.6% had an emergency indication and thus a reason for the dispatching of an emergency device. Accordingly, 17.1% of all ON-Missions by an emergency device were operations with a PTS-indication. In contrast to the PTS, the existence of the 24-h availability of emergency devices played an important role; operations triaged with PTS-indications were performed by emergency rescue vehicles during night hours or on holidays and weekends. A provision of patient transport vehicles could remedy this situation. It was also likely that the frequent incidence of non-urgent patient transportations in main operating periods played a decisive role in the misappropriation of emergency devices. OFF-Missions however were predominantly triaged as urgent operations, which could be explained by EMS-indications and/or lack of prior medical contact. Over-prioritization seemed to be essential at this point. For some emergency diseases, the need for transport did not arise (e.g. unsuccessful resuscitation, death confirmation). Here the disposition rate was also slightly higher to the detriment of emergency devices (+ 7.6%). Patient transport service played a subordinate role, which showed the scheduling of such operations (so-called appointment trips), where the majority of patients have already had contact with a doctor.

### Urban-rural distribution

Almost 3/4 (74.1%) of all ON-Missions were in urban areas, which only accounted for 44.9% of the total population (Fig. 3) [40]. The demand for patient transport services in rural areas was very low for ON- and OFF-Missions (4.9% vs. 2.3%).

Therefore, devices of the PTS are stationed primarily in urban areas. Relatively more OFF- than ON-Missions took place in rural areas (34% vs. 26%), which were almost entirely provided by emergency devices in the case of emergency events (31.7% vs. 21.1%). The use of prehospital services was therefore not significantly determined by the number of inhabitants, but rather other factors played a dominant role. Specific infrastructural institutions such as nursing homes, where elderly and multimorbid people usually lived, were found almost exclusively in cities and call for emergency services more frequently [41–45]. The anonymity of the city, with the missing support of the family and neighborhood, and the population’s increased expectations also caused the number of operations to rise rapidly [46, 47]. In urban areas are also more vulnerable population groups (e.g. homeless people), who often presented themselves as frequent users of emergency services [42, 45, 48–51].

### Job cycle time

On average, the job cycle time of ON- and OFF-Missions differs by 32.5 min, which could be explained by the lack of patient transfer and handover time in OFF-Missions (Fig. 4). Very short job cycle times of 2 s resulted from incorrect dispositions of the EMDC, which have already been received by the dispatched vehicle crew via radio. Long-lasting operations of several hours to almost 24 h are explained by missed sign offs of the emergency device via radio or due to long-distance trips.

### Remuneration

The approach of the applied calculation is very simply structured and does not correspond in its amount to the actual costs, since some cost items were not taken into account. The aim of this work was a rough illustration of the remuneration of prehospital medical service and has no claim to completeness with the aim of follow-up studies in this field. In both, the ON and OFF cohorts, emergency devices (mainly emergency rescue vehicle and emergency physicians’ operational vehicle) generated the most (€ 108,822,272) and recorded the largest number of lost revenues (€ 12,495,564). In general, OFF-Missions were not reimbursed by the funding agencies and that’s why this was referred to as lost revenues. At this point, the different basic rate of the prehospital devices has not been ignored. The number of missions by emergency physicians’ cars was very small, as they have been increasingly replaced by emergency physicians’ operational vehicle and were now used almost exclusively as intensive care transport vehicles for emergency physician-assisted transfers at exclusive locations. Long access distances lead to the large mean. Interestingly, the means of all prehospital devices appeared very homogeneous, with the exception of the above-mentioned vehicle type. The reason for this could be the longer residence time of emergency devices at the scene for the treatment of the patient with shorter transfer times and the shorter residence time of patient transport vehicles with longer transport routes for special examinations.

## Conclusions

This study highlights in particular the increasing use of emergency devices, which is also associated with a progressive misappropriation of these vehicles; especially in the case of OFF-Missions, the EMS records a higher number of operations than PTS; the reasons are a combination of the fact, that emergency operations and their course are not plannable or predictable. From an economic point of view, OFF-Missions are a non-profit business for the service providers; immensely high revenues are missing, but in the present work these are only calculated in a simplified way and thus presented to a lesser extent than it is actually the case. Therefore, better patient management appears to make sense from both sides - the medical and the economic point of view. Practical experience has shown that simple assistance is more time-consuming than normal emergency events, on the one hand because of legal uncertainty in the case of trivial indications and on the other hand because of complex medical care (e.g. unsuccessful resuscitation). In order to be able to make statements about the increased effort of simple assistance, the effective treatment time (= on-site time) between ON- and OFF-Missions should be compared in future studies.

Future studies should also investigate whether and how the generously defined indications of EMS and PTS may have led to higher-quality care for patients. This could be measured by a decrease in hospital admissions that could be avoided by EMS and PTS, shorter hospital stays and better patient outcomes. For specific emergency diseases such as acute stroke and out-of-hospital cardiac arrest, previous studies have shown that a better functional outcome could be achieved by reducing treatment-free time with the help of additional resources [52, 53]. Currently, due to legal uncertainties towards dispatchers and paramedics on the scene, transports to inpatient institutions are increasingly performed after alerting EMS and PTS, which could have been avoided; outpatient, primary care treatment would have been sufficient [54, 55]. Practical experience teaches that such admissions often lead to associated disorders such as nosocomial infections, which increase both hospitalization time and mortality. The legal uncertainty results from German law, which defines EMS and PTS according to its role as a transport company and not as a provider of prehospital emergency medicine, so that remuneration is only paid in case of transport, which creates misaligned incentives in terms of such avoidable admissions [56]. From a socioeconomic point of view, a political rethinking could strengthen and appreciate the position and work of EMS and PTS (remuneration of outpatient missions), provide patients with more goal-oriented care and protect them from overuse, save hospital resources, and thus reduce health care costs.

## Data Availability

The data that support the findings of this study are available from the Department of Civil Protection of the district administration Mainz-Bingen/Germany, but restrictions apply to the availability of these data, which were used under license for the current study and are therefore not publicly available. The Data were made available upon the authors reasonable request and with permission of the Department of Civil Protection of the district administration Mainz-Bingen/Germany. Please contact Dr. Guido Scherer for details.

## Abbreviations

EC: Einsatzcode (in German; corresponds to the operation code)
EM: Einsatzmittel (in German; corresponds to prehospital devices)
EMDC: Emergency Medical Dispatch Centre
EMS: Emergency Medical Service
KL: Krankentransport liegend (in German; corresponds to non-urgent transport in horizontal position)
PTS: Patient transport service
R: Rettungswagen (in German; corresponds to the emergency rescue vehicle)
SQL: Structured query language
